# Vectorial Release of Human RNA Viruses from Epithelial Cells

**DOI:** 10.3390/v14020231

**Published:** 2022-01-25

**Authors:** Sabine Chapuy-Regaud, Claire Allioux, Nicolas Capelli, Marion Migueres, Sébastien Lhomme, Jacques Izopet

**Affiliations:** 1 Department of Virology, CHU Purpan, F-31059 Toulouse, France; nicolas.capelli@uclouvain.be (N.C.); migueres.m@chu-toulouse.fr (M.M.); lhomme.s@chu-toulouse.fr (S.L.); izopet.j@chu-toulouse.fr (J.I.); 2 INFINITy (Toulouse Institute for Infectious and Inflammatory Diseases), INSERM UMR1291, CNRS UMR5051, Université Toulouse III, CHU Purpan, F-31024 Toulouse, France; claire.allioux@inserm.fr

**Keywords:** human RNA virus, epithelium, polarization, release or egress

## Abstract

Epithelial cells are apico-basolateral polarized cells that line all tubular organs and are often targets for infectious agents. This review focuses on the release of human RNA virus particles from both sides of polarized human cells grown on transwells. Most viruses that infect the mucosa leave their host cells mainly via the apical side while basolateral release is linked to virus propagation within the host. Viruses do this by hijacking the cellular factors involved in polarization and trafficking. Thus, understanding epithelial polarization is essential for a clear understanding of virus pathophysiology.

## 1. Introduction

Epithelial cells line all tubular organs and are often the first targets of infectious agents. Their apico-basolateral polarization is due to molecular events that occur throughout their development. These ones modify the cell membrane composition and produce different trafficking routes to and from each pole [1]. These mechanisms can be hijacked by viruses at several stages of their life cycle, resulting in polarized entry and release. While apical or basolateral entry depends mainly on the distribution of virus receptors, virus egress results from the interaction of the viral proteins with intracellular factors. Vectorial release implies a non-lytic process, classically used by enveloped viruses, while particles of quasi-enveloped viruses, which lack peplomers [2], and certain naked viruses, use membranes for non-lytic egress. The resulting extracellular vesicles can contain several virus particles, enabling their en bloc transmission and more efficient virus spreading [3].

This review focuses on studies that analyze the amounts, infectivity and physicochemical characteristics of human RNA virus particles released from both sides of polarized human cells grown in transwells. The most popular models are those of the intestine, lung and liver, and the majority of studies have used primary cells or cell lines polarized in vitro. While organoid models have been developed recently they have rarely been used yet to study the polarized release of viruses. Finally, we indicate the key cellular factors involved in polarized release.

## 2. Characteristics of Polarized Epithelial Cells

**Figure 1:**
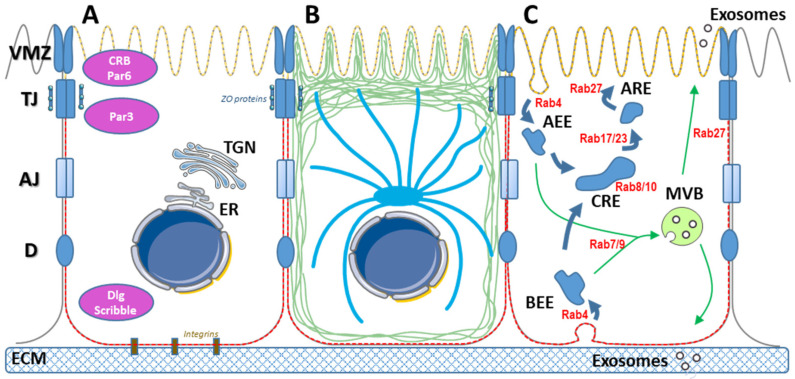
Characteristics of epithelial cells. (**A**) Epithelial cells polarity is maintained by several junctional complexes including the vertebrate marginal zone (VMZ), tight junctions (TJ), *adherens* junctions (AJ), desmosomes (D). These complexes depend on several proteins involved in polarity, including Crumbs and Par at the apical side and disks large (Dlg) and Scribble at the basolateral side. Integrins are main proteins involved in the adherence to the extracellular matrix (ECM). ER: endoplasmic reticulum. TGN: Trans Golgi Network. The apical and the basolateral membranes are enriched in phosphatidylinositol (4,5) bisphosphate [PtdIns(4,5)P2] (yellow hashed line) and PtdIns(3,4,5)P3 (red hashed line), respectively. (**B**) Schematic representation of the actin (green) and microtubules (blue) polarization. ZO proteins ensure the link between junctional complexes and the cytoskeleton. (**C**) Apical and basolateral endocytosis pathways, initiated at apical or basolateral early endosomes (AEE and BEE, respectively) converge to the multivesicular body (MVB) or to the common recycling endosomes (CRE), from which apical recycling endosomes (ARE) can bud. Distinct Rab proteins are involved at each step of these processes.

Epithelia are monolayer or multilayer tissues lining all tubular organs; their cells are attached to a basement membrane by their basolateral side and their apical side is exposed to a lumen or the air. Their planar polarization, with specific cell-to-cell contacts, results in a physical barrier between the two sides of the epithelium. Some epithelial cells form systems that are more complex. For example, the basolateral side of hepatocytes faces the liver sinusoidal endothelial cells and one or more apical sides face bile canaliculi [4]. The compositions of the apical and basolateral membranes of these epithelial cells become very different during differentiation to ensure the proper function of the epithelium [5] (Figure 1).

The integrity of epithelia depends on adhesion between cells that involves several junction protein complexes including tight junctions (TJ), *adherens* junctions, and desmosomes. Tan et al. recently described a distinct polarity domain at the apical side of TJ, the vertebrate marginal zone (VMZ), in Madin-Darby canine kidney (MDCK) cells [6], while Gap junctions are involved in intercellular communication [7]. Conserved polarity modules, Crumbs and Par (apical) and Scribble and Dlg (basolateral) all govern the correct assembly of these junctional complexes [1]. TJ are composed of several transmembrane proteins on the extracellular plasma membrane (PM) side that interact with homologous proteins of adjacent cells. Claudins, occludins and junction adhesion molecules (JAM) are essential components of TJ [8]. On the intracytoplasmic side, several proteins containing PDZ domains, such as *zona occludens* (ZO) proteins, form scaffolds for these complexes and ensure their junction with the cytoskeleton. TJ allow only water, ions and small molecules like sugars to cross the epithelium. They also maintain the different compositions of the apical and basolateral plasma membranes. During development, cell contacts induce the mutual exclusion of proteins dedicated to the apical and basolateral sides, on each side of TJ [9]. Among them, integrins contribute to link the basolateral side to the extracellular matrix [10]. The segregation of phosphoinositides between the poles is also critical for epithelial polarization. Phosphatidylinositol (PtdIns) (4,5) bisphosphate (P2) is concentrated in the apical membrane while PtdIns(3,4,5)P3 accumulates at the basolateral side [11]. PtdIns(4,5)P2 specifically recruits proteins like Annexin-2 and Slp2-a. Annexin-2 binds Cd42, that helps form tight junctions and directs trafficking to the PM [12,13]. These processes are closely maintained and regulated by enzymes that modify phospholipids [12,14,15]. PtdIns(3,4,5)P3 plays a critical role in polarized protein recruitment. Adding exogenous PtdIns(3,4,5)P3 to the apical side of MDCK cells orients basolateral proteins to their apical pole [16]. Actin is also important for maintaining polarity; the actin cytoskeletal network interacts directly with TJ junctional molecules, including ZO proteins [17].

The secretion and trafficking of extracellular vesicles are also polarized. Secreted or membrane proteins are sorted in the trans-Golgi network and orientated towards the appropriate pole [18]. The main drivers of the selective recycling of endosomes to the apical or basolateral plasma membranes during endocytosis-exocytosis are soluble N-ethylmaleimide-sensitive attachment protein receptors (SNAREs) and Rab proteins [19]. Rab4-dependent apical and basolateral early endosomes can both fuse to a common Rab8- and Rab10-positive recycling endosome. Rab11 and Rab17 then play a major role in vesicular trafficking to the apical recycling endosome, while Rab27 ensures their passage to the apical plasma membrane [20,21]. VAMP8/Endobrevin addresses extracellular vesicles to the basolateral side of MDCK cells [22], while Rab27a and Synaptotagmin-Like Protein 2a (Slp-2a) address vesicles to the apical side. Slp-2a then interacts with PtdIns(4,5)P2-rich membrane domains [13]. The compositions of basolateral and apical exosomes differ at the end of this process [23]. Apical exosomes from human colon carcinoma LIM1863 cells are enriched in EpCAM while the basolateral exosomes are enriched in A33 antigen [24]. The apical exosomes of MDCK cells polarized in culture are enriched in HSP70, GPR5C and CD63 while the basolateral exosomes have more TSG101, CD81 and CD9 [25,26]. This depends on the cell type since CD81 is enriched in the extracellular vesicles secreted from the apical side of proximal tubular epithelial cells [27].

## 3. Experimental Systems for Studying Vectorial Virus Release

Polarized cells must be grown in transwells in order to study the release of virus particles from both sides of these cells, as this provides them with basolateral and apical sides. Differentiation can be promoted by coating the membrane with extracellular matrix (ECM) components like collagen or gelatin, or the ECM produced by the Engelbreth-Holm-Swarm (EHS) mouse sarcoma, commercialized as Matrigel [28]. ECM components help induce cell polarization [1]. These inserts are maintained in culture with medium added to the basal side. The apical side is usually covered with medium, which may be identical to or different from the basal medium, depending on the experiment. An air-liquid interface can be generated with no medium added above the insert to mimick respiratory epithelia [29]. Virus particles released from the apical side of these systems can be recovered by washing [30]. Studies on virus propagation in polarized cells often use replicative viruses that can propagate in culture. Comparison of the amounts of viral markers on each side of transwells is usually the result of these successive infection cycles. This must be considered when interpreting the results, particularly if there is no difference between apical and basolateral infections. 

MDCK cells or their derivatives are widely used because they are readily polarized in culture [31]. Cell lines from organs of interest or their subclones are also used: A549 for lung, Caco-2 for intestine, and Huh-7 or HepG2 for liver. These cell lines are readily available and long-lived but they do not reproduce all the characteristics of the primary organ. Intestinal and respiratory primary cells are frequently used to reproduce a polarized 2D-epithelium in culture [32,33]. The liver epithelium is particular in that it has a 3D architecture [4]. However, 2D systems have been set up to study traffic and transcytosis [34]. Polarized cells can also be differentiated from adult or pluripotent stem cells [35]. Human stem cell-derived hepatocyte-like cells (HLCs) have been polarized on inserts to reproduce the hepatocyte phenotype [36]. Hepatocyte can be polarized by adding dimethylsulfoxide (DMSO) to the culture medium [37,38]. Organoids are cellular systems that allow to reproduce the characteristics of the corresponding adult tissues including their multicellular composition [39].

There are several ways to check epithelium polarization. The transepithelial electrical resistance is increased as a result of the barrier generated during polarization [40]. Labeling TJ proteins show relatively straight, continuous lines that connects tricellular contact points. Ruffles in TJ result from the interaction of claudins with the cytoskeleton and can alter permeability, while spikes in TJ are involved in intercellular communication [41]. Functional cell polarization can also be evaluated by measuring secreted components. For example, hepatocytes secrete albumin from their basolateral side and bile acids from their apical side [42]. Polarized secretions by organoids can be studied by reversing them or laying then on transwells [43].

## 4. Exit Poles and Production of RNA Virus Particles

### 4.1. Polarized Release of RNA Viruses Infecting Mucosal Epithelia and Triggering Local Related Symptoms

Viruses transmitted by the respiratory or oral routes first encounter the apical side of the corresponding epithelium. Coronaviruses (CoV) enter epithelial cells via the apical membrane, following interaction of the Spike protein with a peptidase receptor. HCoV-NL63, SARS-CoV and SARS-CoV-2 all interact with angiotensin-converting enzyme 2 (ACE2), MERS-CoV interacts with dipeptidylpeptidase-4 (DPP4), and HCoV-229E with aminopeptidase N (APN) [44]. CoV entry is independent of the peptidase activity but could be linked to enrichment of these enzymes on the apical side, in parallel with their low affinity for their natural ligand [45]. Most the coronaviruses infecting airway epithelial cells are released from the apical side [44]. SARS-CoV is mainly released from the apical side of human bronchial epithelial Calu-3 cells in transwell culture systems [46]. SARS-CoV-2 can infect the ciliated and goblet cells, in a human airway epithelium (HAE) cultured at an air-liquid interface (HAE-ALI) and is released from the apical side. The virus has a cytopathic effect, with cell fusions, destruction of tight junctions, and disorganization of cilia [30]. Uninfected basal cells then proliferate to regenerate the epithelium and support a persistent infection [47]. In severe COVID-19, infection of endothelial cells results in virus leakage across the inflamed epithelium rather than the basolateral release of virus particles [48]. MERS-CoV has been shown to infect Calu-3 cells by both sides. While apical infection is more efficient, and infectious particles are preferentially released apically, there is substantial particle release from the basolateral side [49]. Similar results have been obtained with polarized colon Caco-2 cells. This could explain why MERS-CoV is more likely than other CoV to disseminate in its host [50]. Electron microscopic studies showed that influenza A virus (IAV) buds from the apical side of cellular microvilli in differentiated human airway epithelial cells [51]. IAV can infect both sides of porcine tracheal or bronchial epithelial cells at an air-liquid interface, leading to the apical release of virus particles and the loss of cilia. However, the permeability and barrier functions of the epithelium were not altered during the eight-day experiment [52]. The M2 protein of IAV, a viroporin, plays a major role in this apical release. Adding ectopic M2 protein to the basolateral membrane or the endoplasmic reticulum of human nasal epithelial cell (hNEC) cultures inhibited virus production [53]. The cytoplasmic domain of the envelop glycoprotein is a key determinant of its transport to the membrane: replacing the cytoplasmic domain of the vesicular stomatitis virus (VSV) G protein by that of IAV hemagglutinin (HA) changes its localization from the basolateral to the apical membrane [54]. Studies on polarized MDCK or Calu-3 cells grown on inserts show that the negative single strand RNA Mumps virus (MuV, Paramyxoviridae) infects both sides of cultures with similar efficiency but is mostly released from the apical surface, at which its N and M proteins accumulates [55].

However, RNA viruses transmitted by airway or orally do not always preferentially infect the apical side of an epithelium. Rotavirus, a naked double strand RNA virus (Reoviridae), is mainly responsible for gastroenteritis in children. It infects polarized porcine small intestine cells preferentially via the basolateral side but is mainly released from the apical side before cell lysis [56]. Measles virus first infects alveolar macrophages and dendritic cells via its receptor, CD150/SLAM. These cells can then deliver the virus to epithelial cells via their basolateral side [57]. While Measles virus particles can be released from the apical side of human airway epithelium cells [58], recent studies indicate that this release is not fully efficient. Clusters of highly infected cells become dislodged from the epithelium, which could explain why they are highly infective when they are ejected in respiratory aerosols and droplets [59].

### 4.2. Polarized Release of RNA Viruses Infecting Mucosal Epithelia and Triggering Distant Symptoms

Other RNA viruses acquired by the oral or respiratory routes replicate in the corresponding epithelium without evident associated clinical signs and then gain access to secondary organs, with clinical manifestations reflecting this secondary infection. Hepatitis A and E viruses are naturally transmitted orally, but the liver is their major target organ. While virus particles propagate in the environment as naked infectious particles, they are released from cells and circulate in the host as lipid-associated quasi-enveloped particles that lack virus glycoproteins to interact with a cognate receptor. Lipid-associated particles are less infectious than naked particles but can still infect new cells [60,61]. HAV and HEV are transmitted orally and can infect intestinal cells. HAV infects polarized Caco-2 cells in culture mostly via the apical side, and is preferential release apically [62,63]. Primary intestinal cells polarized in Matrigel-coated transwells and infected with HEV via the apical side preferentially release virus particles apically, with a small fraction released basolaterally [64]. For both HAV and HEV, the low proportion of infectious virus particles released at the basolateral side could reach the liver in vivo via the portal vein, and so infect hepatocytes. Clones of HepG2 human hepatocarcinoma cells line were selected for their ability to become polarized in culture. The HepG2-N6 clone is infected by HAV preferentially by the basolateral side [65]. The HepG2/C3A/F2 clone is efficiently infected by HEV apposed at the basolateral side [66]. These observations are consistent with the natural route of these viruses during infection: after apical infection of intestinal cells, virus particles released at the basolateral side could reach the liver via the bloodstream and infect hepatocytes via their basolateral membrane. Once infected, HepG2-N6 cells released HAV preferentially via the basolateral side [65] while the HepG2/C3A/F2 clone preferentially released infectious quasi-enveloped HEV particles apically [66]. HEV expresses ORF3, a small protein with functions similar to the M2 viroporin of IAV [67]. Palmitoylated ORF3 becomes embedded in the membrane of multivesicular bodies, which drives the export of newly assembled virions [68]. ORF3 is necessary for the apical release of HEV particles from HepG2/C3A/F2 cells [69]. Stem cell-derived hepatocyte-like cells also release most HEV particles apically. These cells produce more bile salts than HepG2/C3A/F2 cells and these could strip off the quasi-envelope to give more infective particles [36]. Orally transmitted viruses such as enteroviruses propagate from the gastrointestinal entry site to secondary organs, such as the heart or the central nervous system [70], using several receptors located at the TJ or on the apical side of polarized cells [71]. Phosphatidylserine-enriched large vesicles containing several infectious particles undergo autophagy-dependent release upon infection [72], thus potentiating the spread of the infection [73]. The vectorial release of enteroviruses is mainly apical, as is that of poliovirus from polarized Caco-2 cells [74], that of coxsackie B1 from a human gut-on-a-chip microfluidic device [75], and that of parechoviruses from human airway epithelia [76]. These viruses can bypass the innate immune response of the initially-encountered epithelium [71]. It has been shown that endothelial cells of the human choroid plexus papilloma (HIBCPP) are more sensitive to basolateral infection (than apical) by echovirus-30, one of the enteroviruses responsible for outbreaks of meningitis [77]. This links the apical release from the digestive tract to the basolateral entry into the target organ.

Some RNA viruses acquired by the respiratory or oral routes are highly pathogenic and are rapidly disseminated from their primary infection site. Old World arenaviruses are naturally transmitted by ingestion. These bisegmented ambisense RNA viruses, such as the Lassa virus (LASV), infect Caco-2 cells preferentially apically and are released both apically and basolaterally. Apical release is more efficient after an apical infection. The recombinant strain ML-29 composed of the MOPV L segment, a non-pathogenic relative of LASV, and the S segment of LASV, cannot leave the cell basolaterally. This should prevent intra-host dissemination, which makes it a vaccine candidate [78]. The New World arenavirus, Junin virus, is preferential released apically from both polarized Vero C1008 and MDCK cell lines [79]. Nipah virus (NiV) belongs to the genus Henipavirus (Paramyxoviridae); it is transmitted via the respiratory route by airway secretions and urine. Its receptor, ephrin-B2/-B3, is located on both sides of polarized MDCK cells and infection occurs whatever the inoculated side of cell culture on inserts. Lamp et al. showed that the M protein drives virus assembly and release via the apical membrane. NiV F and G proteins are initially targeted to the basolateral side and induce cell fusion while the M protein is produced in the host cytoplasm. Cell fusion is followed by the apical accumulation of M, F and G proteins, leading to the apical release of NiV particles. Finally, the integrity of the epithelial barrier is lost, allowing the basolateral release of NiV and its dissemination in its host [80]. Marburg virus (MARV), a causative agent of severe hemorrhagic fever, buds preferentially from the basolateral side of MDCK as well as hepatocytes, which is consistent with the systemic dissemination of infectious particles in its host [81].

### 4.3. Polarized Release of RNA Viruses Unable to Cross Mucosa but Subsequently Infecting Epithelia

Many viruses can infect epithelial cells in experimental systems or in their host organism even if they cannot reach or cross the intestinal or respiratory barriers. The hepatitis C virus (Hepacivirus) is mainly acquired by contact with blood after percutaneous injection. Particles of HCV strain JFH1 are released preferentially from the basolateral side of infected polarized HepG2-CD81 cells regardless of the side of infection [82], as might be expected because the HCV replication cycle hijacks the lipoprotein pathway and follows the same release pathway [83]. The genus Flavivirus includes several major arboviruses, Zika virus (ZIKV), Japanese encephalitis virus (JEV), West Nile virus (WNV) and Usutu virus (USUV), all infecting the human upper respiratory tract epithelium without altering its barrier function. All their virus particles are released both apically and basolaterally, but ZIKV and JEV particles are mainly released apically in this model [84]. This could explain why JEV can be transmitted without vectorial transmission between pigs and ZIKV between guinea pigs [85,86]. WNV infecting polarized Vero cells enters and is released mainly apically [87], as is ZIKV in Caco-2 cells [88]. ZIKV also infects a wide range of tissues, resulting in ZIKV proteins becoming colocalized with the apical proteins β-IV tubulin and Muc5A [89]. ZIKV can also infect polarized human brain microvascular endothelial cells (HBMEC); it is released from both side of these cells [90], which shows that vectorial virus release may depend on the cell-type. Infectious Dengue virus (DENV) particles are released in equal amounts from both sides of MDCK cells regardless of the side of infection [91]. Chikungunya virus (CHIKV), an Alphavirus (Togaviridae) is also transmitted by a mosquito bite. CHIKV enters and is released from polarized HBMEC and Vero C1008 cells preferentially apically [92]. VSV, which is seldom pathogenic for humans, preferentially buds from the basolateral surface of polarized human epithelial T84 cell line (derived from a colon carcinoma); this release is driven by its G protein, which accumulates on the basolateral side of host cells [93].

**Figure 2:**
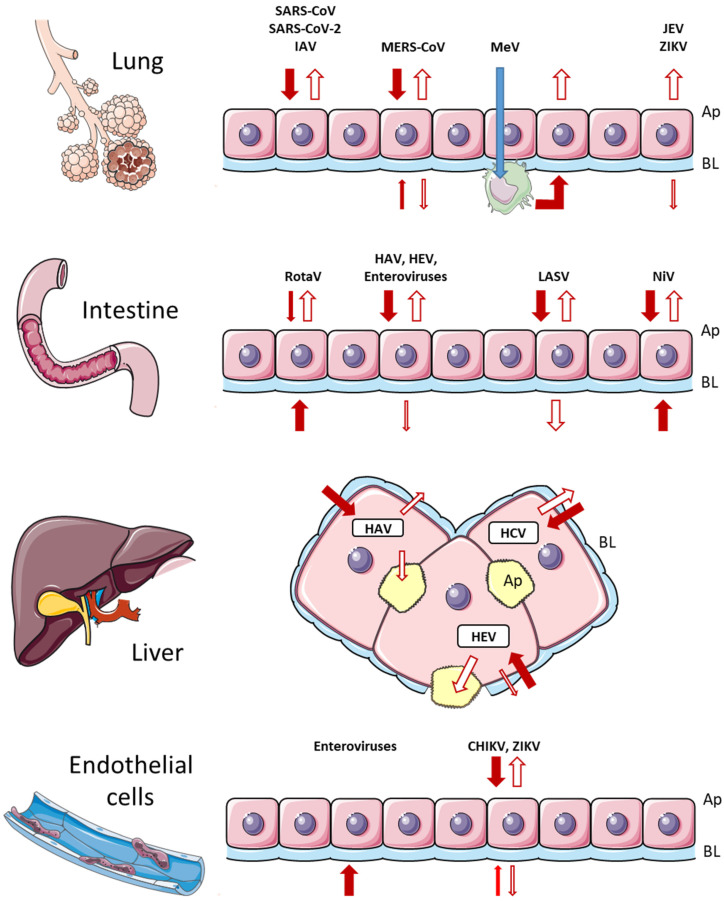
Entry and release of different viruses in epithelial cells. Entry is indicated by a full arrow and release by an open arrow. Thick and thin arrows represent the main and minor routes of traffic, respectively. Details are indicated in the text. Ap, apical side; BL, basolateral side.

In summary, most viruses that infect the mucosa leave the cells mainly apically, while basolateral release is linked to virus propagation in the host. The entry and egress of viruses infecting internal epithelia such as the endothelium or liver are specific to each virus (Figure 2).

## 5. Cellular Mechanisms of Polarized Virus Egress

### 5.1. From the Endoplasmic Reticulum (ER) and the Virus Assembly Sites to the Recycling Endosomes

Infection by an RNA virus induces rearrangement of host cell membranes, mainly ER and Golgi, in order to build platforms for replication [94]. Virus proteins leave the Golgi compartment and become concentrated at the basolateral or apical pole [95]. However, some viruses exploit distinct organelles: the enterovirus 3A protein binds to the Arf1 GTPase and its guanine nucleotide exchange factor GBF1 in the ER membrane, leading to local recruitment of phosphatidylinositol-4-kinase IIIb. The resulting enrichment in PtdIns4P facilitates the virus polymerase binding and initiates replication in newly-formed replication organelles [96]. Studies on polarized Caco-2 cells inoculated with simian rotavirus strain RRV showed that the virus particles never reach the Golgi apparatus but move directly from the ER to the apical membrane [97]. Intermediate compartments generated during coronavirus infection could bypass the Golgi apparatus and be brought by Rab11 to the recycling endosomes [98]. It has been shown that coronavirus can be secreted apically from MDCK cells in the presence of Brefeldin A, which blocks ER to Golgi traffic [99]. 

### 5.2. Rab GTPases

Rab proteins are essential for the biogenesis of infectious virus particles as they are involved in the trafficking of viral proteins and RNA to the correct sites of assembly and budding. Several respiratory viruses use Rab11, which is involved in the traffic from common to apical recycling endosomes. Respiratory syncytial virus (RSV) assembly in polarized MDCK cells is dependent of Rab11a and its interacting protein FIP2 [100]. The M and N proteins of MuV are not apically distributed in polarized MDCK or Calu-3 cells having a dominant negative form of Rab11. Studies on nocodazole-treated cells have shown that functional microtubules are necessary for the correct Rab11-dependent addressing of viral proteins to the membrane [55].

Rab11a is also colocalized with the L protein of MeV. And host cells with a dominant negative mutant of Rab11a do not address MeV particles to the apical membrane [101]. Rab 11 is also needed for the transport of Sendaï and Parainfluenza viruses to the plasma membrane [102], and the transport of Andes Hantavirus [103]. Hendra virus (HeV) M and F proteins converge into Rab11-positive endosomes, making them a preassembly compartment for virus particles [104]. The IAV nucleoprotein remains in the perinuclear zone of host cells transfected with a double negative mutant of Rab11, and does not accumulate in vesicles close to the plasma membrane. This results in the release of significantly fewer infectious IAV particles [105]. Moreover, IAV infection causes the endoplasmic reticulum containing the viral nucleoproteins to swell and recruits Rab11 for the export of newly-formed irregular coated vesicles to the plasma membrane [106]. Rab11a is necessary for the correct distribution of the eight ribonucleoproteins in the newly assembled virus particles [107]. IAV hemagglutinin also interacts with Rab17 and Rab23 in a cholesterol dependent manner, suggesting its Rab17- and 23-dependent association to lipid rafts [108]. Rab27a colocalizes with the capsid ORF2 protein of HEV at the apical side of HepG2/C3A/F2 cells [66]. A lack of Rab27a reduces the propagation of several RNA viruses, including human and rat HEV in human hepatocarcinoma cells PLC/PRF/5 [109,110], human parainfluenza virus type 2 (hPIV-2) in HeLa cells [111], and rabies virus in Vero cells [112]. 

Silencing of Rab9 showed that it is involved in the propagation of MARV in Vero cells [113] and of HCV in the Huh7.5 cell line [114]. Rab9 is involved in the trafficking of vesicles to the MVB, which suggests that these two viruses released by the basolateral side use the exocytosis pathway. However, a traffic route involving actin and Rab11 has also been described for MARV [115], highlighting that different Rabs may be used by viruses in their life cycle.

### 5.3. Cytoskeleton

During viral egress, viral elements have to pass through the dense cytoskeleton structures maintaining the cell architecture [116]. Studies on the role of actin and microtubules in virus-infected polarized cells have shown that the apical membrane is the preferred side for the release of human Parainfluenza virus type 3 (PIV3) particles from polarized human A549 cells. Disruption of the actin microfilaments with cytochalasin D has no effect on PIV3 release, while release is blocked by nocodazole, which inhibits microtubule polymerization, in the twelve hours following the blocking of protein synthesis with cycloheximide [117]. The egress of Rift Valley fever virus (RVF) from polarized Caco-2 cells is only moderately apically polarized. The microfilaments and microtubules of infected cells are disrupted, which could help the virus particles reach the basolateral side for egress [118]. The rotavirus protein VP4 binds to the actin network at the apical pole of infected polarized Caco-2 cells. This is an active process, since inhibiting actin treadmilling with jasplakinolide leads to the loss of preferential apical virus particle release [119]. A comparative study of RSV and human Metapneumovirus (hMPV) infections in human airway epithelia showed that both viruses infect cells via the apical side. While RSV is efficiently released from the apical side, hMPV is not; it forms filamentous actin-based extensions that suggest cell-to-cell transmission [120]. MeV can also hijack the actin network of well-differentiated primary cultures of human airway epithelial cells and so propagate the infection horizontally; RNPs move along the circum-apical F-actin ring. This is accompanied by cell fusion but leads to the formation of infectious centers in which the epithelium remains polarized [121].

### 5.4. Lipids

It has long been known that the lipid composition of enveloped viruses depends on the cell budding membrane. VSV buds at the basolateral pole and fowl plague virus (FPV) buds at the apical pole of infected polarized MDCK cells and they have different phospholipid contents [122]. The IAV hemagglutinin (HA), neuraminidase (NA) and nucleoprotein (NP) are driven to the host cell apical membrane where they are associated with lipid rafts [123]. HA must be directly associated with cholesterol for it to function fully [124]. Rotavirus VP4 spike protein associates with lipid rafts in both polarized intestinal Caco-2 cells and unpolarized renal MA-104 cells. Differences in the virus egress from these two host cells are due to differing lipid raft compositions; Caco-2 cell rafts have more neutral glycolipids with more hydroxylated chains than MA-104 cell rafts. The virus leaves the apical side in Caco-2 cells by budding and leaves MA-104 cells by lysis [125]. PI and PI kinases and PI phosphatases play a major role in the building and maintenance of cell polarity. The HCV core protein moves to the basolateral membrane of polarized MDCK cells where it blocks the synthesis of the major regulators of polarity, Scribble and Dlg1. This results in disorganization of focal contacts and actin and reduced activation of the actin-regulating Rho GTPase, Rac1. This phenotype is due to the decrease in PtdIns(3,4)P2, the PI that recruits Dlg1 in the basolateral membrane, and in SHIP2, a phosphatase that converts PtdIns(3,4,5)P3 to PtdIns(3,4)P2 [126].

### 5.5. Tight Junctions

Tight junctions maintain the integrity of polarized epithelia. Some viruses can alter the barrier function of the epithelium they infect, cross it, and spread within the infected host. This requires modification or degradation of TJ. Polarized Caco-2 cells infected with rotavirus have altered TJ with disorganized occludin [127]. The glycoproteins and fusion protein in enveloped viruses make them fusogenic. Fusion of NiV-infected cells leads to loss of TJ [80]. DENV infection modifies the subcellular distribution of claudin-1, ZO-1 and ZO-2 proteins and the cleavage of occludin in MDCK cells. The resulting increase in 70 kDa FITC-Dextran passing across the cell layer shows that the transepithelial barrier is defective [91]. The differentiated blood brain barrier is also altered as early as two days after infection with JEV, as shown by the decreased trans-endothelial electrical resistance [128]. Several RNA viruses produce proteins that can bind to the PDZ domain of adapter proteins involved in TJ integrity, leading to disruption of the epithelium [129]. For example, SARS-CoV-1 and SARS-CoV-2 E proteins bind to the PALS1 PDZ domain of the Crumbs apical complex leading to loss of epithelial barrier function [130]. The interaction of viral proteins with PDZ-containing proteins also influences cell immune responses, such as the interferon response [131].

## 6. Conclusions

RNA viruses can hijack the pathways of virus release from polarized epithelial cells. Mucosal epithelial cells are main replication and release sites of many viruses. They distribute infectious virus particles toward one or both poles of these primary entry sites, which determines whether the virus spreads within and/or between hosts. Tools that reproduce cell polarization in vitro have been developing from primary cell cultures and stem cells in order to study these phenomena. Transwells remain the main system for selectively recovering apical and basolateral virus particles and determining their amounts and properties. Organoids, the newest models, have several attractive features. They can be reversed or open and laid on transwells to gain access to apical secretions. They can also be used to study cellular trafficking and the interaction of viral processes with the innate response, and they reflect the multicellular nature of the original tissue. These systems are useful tools for analyzing the trafficking that is a fundamental component of the virus cell cycle and propagation in polarized epithelial cells. Further studies analyzing the compositions of the virus particles released from each pole will provide a clearer understanding of virus pathophysiology. Key questions that remain to be addressed are: (i) to what extent do viruses use the pre-existing cellular routes of polarized trafficking? (ii) which characteristics of the apical and basolateral virus particles are related to their propagation in the infected host or between hosts?
